# Melatonin and Brain

**DOI:** 10.2174/157015910792246263

**Published:** 2010-09

**Authors:** Dun-Xian Tan

Melatonin as a natural occurring free radical scavenger and an inducer of antioxidant enzymes has been well documented in thousands of publications within the last decade. Melatonin is no longer exclusively classified as a neurohormone since melatonin has been identified in bacteria, fungi, algae and plants. Likewise, endogenously-produced melatonin is no longer the only source in the body since melatonin is also derived from the diet when vegetables, fruits, cereals, herbs, olive oil, wine or beer are consumed. One important characteristic of melatonin is its permeability into the brain. It readily passes through the blood-brain-barrier and accumulates in the central nervous system at substantially higher levels than exist in the blood. As a result, this molecule exhibits strong neuroprotective effects, especially under the conditions of elevated oxidative stress or intensive neural inflammation.

This volume contains review articles that summarize the newly-described actions of melatonin in the brain. The central nervous system of vertebrates, in addition to the pineal gland, produces melatonin at several sites including in astrocytes, other glia, some neurons and in the meninges. These novel findings point out new aspects regarding the physiological actions of melatonin in the brain.

The neuroprotective effects of melatonin have been tested in many different animal models. These include models of Parkinson’s disease, Alzheimer’s disease, stroke and chemical toxicities. The outcome of these studies provides highly promising evidence that melatonin will prove to be very important in reducing loss of neurons and glia under pathophysiological conditions. The results of clinical trials performed within the last half decade support this conclusion. Some of the major actions of melatonin are mediated at the mitochondrial level such as the action of free radical avoidance.

In addition to melatonin, it is now clear that metabolites of this indoleamine are also significant in executing some the effects initially thought to be exclusively a function of the parent molecule. These important metabolites include especially cyclic-3-hydroxymelatonin, AFMK and AMK. Melatonin as well as these previously-mentioned metabolites are found also in mitochondria, which may be a major site of melatonin’s actions.

These subjects are summarized in the chapters included in this volume. I am grateful to the authors of these contributions for conscientiously reviewing their respective literature in an objective manner.

